# Pilot study on benzene exposure risks in healthcare workers: a combined socio-demographic and computational toxicology approach

**DOI:** 10.3389/fpubh.2025.1634439

**Published:** 2025-12-04

**Authors:** Yuan Zhang, Waqar-Un Nisa, Audil Rashid, Aansa Rukya Saleem, Luqman Riaz, Saima Kalsoom, Abubakr M. Idris, Guo Yu, Habib Ullah

**Affiliations:** 1 University Engineering Research Center of Watershed Protection and Green Development, Guangxi, Guilin University of Technology, Guilin, China; 2 Guangxi Nature Museum for Earth Memory, Lipu, China; 3 Center for Interdisciplinary Research in Basic Sciences (SA-CIRBS), International Islamic University, Islamabad, Pakistan; 4 Department of Botany, University of Gujrat, Gujrat, Pakistan; 5 Department of Earth and Environmental Sciences, Bahria University, Islambad, Pakistan; 6 Department of Environmental Sciences, Kohsar University Murree, Muree, Pakistan; 7 Department of Chemistry, PMAS-Arid Agriculture University, Rawalpindi, Pakistan; 8 Department of Chemistry, College of Science, King Khalid University, Abha, Saudi Arabia; 9 Research Center for Advanced Materials Science (RCAMS), King Khalid University, Abha, Saudi Arabia; 10 Innovation Ceneter of Yangtze River Delta, Zhejiang University, Zhejiang, China

**Keywords:** benzene, health care workers, molecular docking, Rawalpindi, risk assesment

## Abstract

**Introduction:**

Healthcare professions involve frequent exposure to harmful chemicals such as benzene, particularly in environments with suboptimal hygiene, posing significant occupational health risks. This pilot study aimed to assess benzene exposure among healthcare workers in Rawalpindi, Pakistan.

**Methods:**

Blood samples were collected from 51 healthcare workers, including laboratory technologists, paramedics, nurses, and housekeeping staff, at six major hospitals. Benzene concentrations were analyzed using high-performance liquid chromatography (HPLC). Socio-demographic data were obtained via questionnaires. Molecular docking was performed to explore benzene’s neurotoxic potential.

**Results:**

The mean benzene concentration in blood was 0.79 ppm. Healthcare worker category, body mass index (BMI), and job duration significantly predicted benzene levels. Prolonged work hours correlated strongly with benzene body burden (*r* = 0.65, *p* < 0.01). Molecular docking indicated weak but significant binding of benzene with the MAO-B enzyme.

**Discussion:**

These preliminary findings highlight the occupational hazards benzene poses to healthcare workers and suggest the need for improved workplace safety and biomonitoring. The study briefly discusses gender and socio-labor factors, emphasizing institutional responsibilities to ensure safe working conditions. Larger-scale studies are warranted to validate these pilot observations.

## Introduction

Healthcare settings are environments where the prevalence of infectious disease pathogens is high, presenting significant risks to patients, staff, and the broader community if environmental health standards are insufficient (1). Healthcare workers (HCWs) face numerous hazards, including exposure to infectious diseases, unsafe patient handling, hazardous chemicals, and inadequate sanitation (1, 2). These risks are further amplified by poor infrastructure and overcrowding, which impede effective infection control measures. In Pakistan, the majority of healthcare workers in both government and private hospitals continue to work under substandard occupational conditions. Due to their exposure to blood, body fluids, and contaminated surfaces, these workers are at significant risk of acting as vectors for infectious diseases, including HIV, Hepatitis B and C, tuberculosis, and COVID-19 (2–4). Unsafe practices, such as the reuse of non-sterile needles, further exacerbate these risks. The elevated risk of infection among HCWs not only jeopardizes their health but also undermines their ability to deliver care, potentially diminishing the overall capacity of healthcare services (5). Moreover, poor working conditions carry significant economic consequences, as HCWs may be forced to purchase their own cleaning supplies and perform additional tasks without compensation (6, 7). Addressing these challenges is essential to safeguard HCWs and prevent the transmission of infections within healthcare settings in Pakistan.

There is a link between poor environmental hygiene in healthcare settings and direct transmission of microorganisms, causing hospital-acquired infection (8). Apart from pathogenic infections, workers in different occupations are also exposed to a number of chemical contaminants (9). The route of exposure to organic pollutants is primarily through inhalation and skin (10). Benzene and toluene are some of the examples of those chemicals to which healthcare workers are chronically exposed (11). Benzene is one of the most widely used industrial chemical agents. Its exposure is known to cause a wide range of hematopoietic disorders, such as plastic anemia (12). In case of personal exposure, benzene levels may exceed in indoor environments than in outdoor environments, and many studies have confirmed this (13). It can result in various disorders due to high concentration, causing disturbance in the central nervous system, headache, dizziness, respiratory arrest, and ultimately leading to death. Chronic benzene exposure causes aplastic anemia (9). There are a number of factors that account for the extent of occupational exposure, e.g., the amount and times the exposure occurs and the extent of practicing the self-protection (14). Exposure to hazardous drugs in health care settings has been addressed by National Institute for Occupational Safety and Health (NIOSH). It was meant for healthcare workers to take protective measures against such exposure (POEA n.d). HCWs, in general, and nurses, in particular, face occupational exposure to organic solvents in routine, e.g., acetone, chloroform, benzene, and methanol. These chemicals are commonly found in laboratory reagents and sterilizing agents (15). Molecular docking analysis was used to study the interaction between the exposed contaminant and its toxicological effects on the biological target. Molecular docking analysis was studied by using the MOE software 2018. MOE is a software system designed by the Chemical Computing Group to support bioinformatics, cheminformatics, molecular modeling, virtual screening, Quantitative Structure-Activity Relationship (QSAR), Structure-Based Drug Design (SBDD), and Ligand-Based Drug Design (LBDD) and can be used to figure new applications based on scientific vector language (SVL) (MOE) (16). The healthcare settings in government hospitals of Pakistan are substandard. This study aimed to focus on future health implications for exposed workers. Even though infectious hazards in healthcare settings have been studied extensively, little is known about healthcare workers’ occupational exposure to chemicals like benzene, especially when it comes to combining exposure assessment and molecular toxicology to clarify potential health risks. The current study was planned to investigate the exposure of health care staff to organic solvents (benzene) in a healthcare setting, an area of study is neglected and rarely addressed.

## Materials and methods

### Study area

The study was conducted in six major hospitals of Rawalpindi city, including both private and government hospitals. Hospitals selected for this study were Holy Family Hospital; Rawalpindi General Hospital (Benazir Bhutto Shaheed Hospital); District Headquarter Hospital (DHQ), Rawalpindi; Hearts International Hospital, Rawalpindi; Madina Medical Center; and Ahmed Medical Complex, Rawalpindi.

### Sample collection

Fifty-one blood samples were collected from the healthcare workers from these hospitals, i.e., from staff nurses (*n* = 17), lab workers (*n* = 13), and housekeeping staff (*n* = 21). The number of blood samples from each hospital was 10, each from Rawalpindi General Hospital, Rawalpindi; District Headquarter Hospital, Rawalpindi; Hearts International Hospital, Rawalpindi; and Ahmed Medical Complex, Rawalpindi. Seven blood samples were collected from Holy Family Hospital, and five blood samples were collected from Madina Medical Center, Rawalpindi. Blood samples were collected from an antecubital vein. A 10-ml disposable plastic syringe with a 21-gage needle was used for each subject for blood testing, and 6 mL of blood was transferred into a plain vacutainer tube containing no anticoagulants. Samples were centrifuged for 20 min at 3,000 rpm, and the serum was separated. Data on demographic aspects were also collected from each subject with the help of a questionnaire, which included information on age, gender, education, height, weight, body mass index (BMI), smoking habits, occupational history, as well as, medical history of each worker.

### Analysis of blood samples

Quantitative analysis of blood samples for benzene was carried out in the experimental laboratory of PMAS–Arid Agriculture University, Rawalpindi. The extraction was carried out using the protocol reported by Al-Daghri (17). Prepared samples were analyzed using High-Performance Liquid Chromatography (HPLC) SPD 10A VP– Shimadzu equipped with an RP-C18 column, auto-injector (Shimadzu SIL-10 VP) UV/VIS Detector (Shimadzu SPD- 10AVP, at 254 nm). The mobile phase was acetonitrile under varying concentrations with water (UV-HPLC PAI-ACS Panreac, *M* = 18.016) at a flow rate of 1 mL min^−1^ in isocratic elution mode (P.I = 8.32) at ambient temperature. Blood benzene in the samples was identified on the basis of respective retention times and quantified on the basis of respective peak areas. The concentration of benzene in the blood sample was calculated with the help of the following formula.


$$\begin{array}{l} \text{Concentration of unknown sample}\;(\mathrm{ppm})\\ =\frac{\text{Conc}.\text{of standard}\;(\mathrm{ppm})\times \text{Area}\;(\text{sample})}{\text{Area}\;(\text{standard})} \end{array}$$

### Quality control parameters

Chemicals used for the extraction of Benzene and sample cleanup from blood were HPLC or analytical grade, i.e., n-hexane (Merck, No: 843, 95% pure), diethyl ether (Assay-GC 99.5% pure, Riedel-de Haen), sodium sulfate anhydrous (99% pure; Panreac), and methanol (Assay-GC 99.7% pure; Riedel-de Haen). Final prepared samples were stockpiled in acetonitrile (MW 41.05 HPLC grade; Sigma Aldrich) and also used as the mobile phase with water (UV-HPLC PAI-ACS Panreac, *M* = 18.016). Benzene standard (Assay-GC 99.7% pure; Riedel-de Haen) was purchased from BDH Laboratory Supplies. Stock was prepared in acetonitrile from which calibration standards were prepared by serial dilutions. Prepared standards were run a number of times to optimize the signal-to-noise ratio. The limit of detection (LOD) and the limit of quantification (LOQ) were determined by the signal-to-noise ratio (S/N) method. Standard and sample recovery were maximized to ensure quality control so that, after every 10 samples, one standard and one duplicate sample were run to validate the result of the HPLC analysis.

### Molecular docking

**Figure 1:**
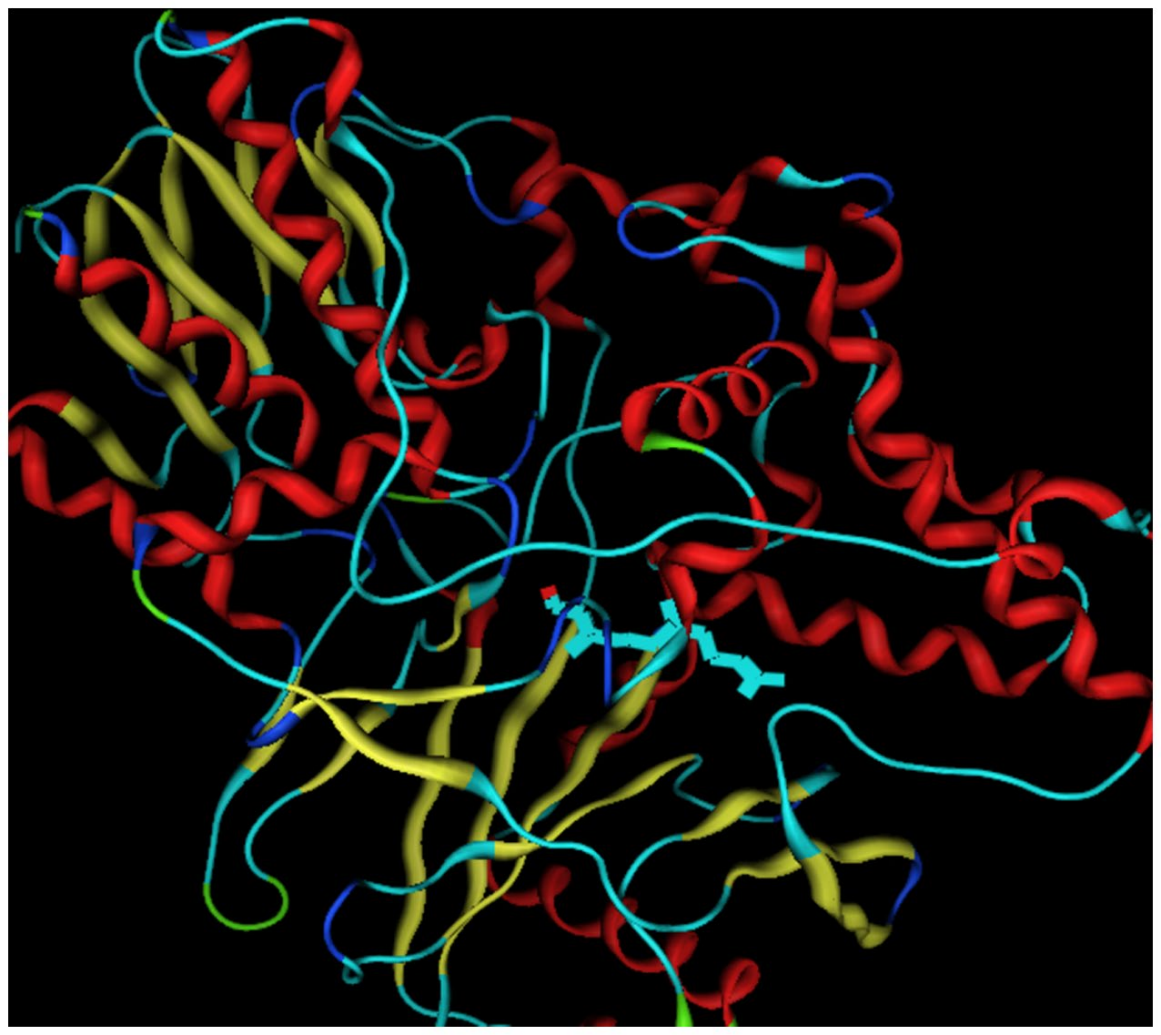
A 3D view of antidepressant target MAO B (PDB id: 2BK3).

The molecular docking tool, MOE 2018, was used for investigating the binding effect of benzene with one of the antidepressant targets, MAO-B. The crystal structure of MAO B was obtained from the protein data bank (PDB ID: 2BK3)^1^. The structure of the complex was prepared using the builder tool of MOE. The energy of the protein molecule was minimized using the energy minimization algorithm of the MOE tool. The following parameters were used for energy minimization; gradient: 0.05; force field: MMFF94X + Solvation; chiral constraint: current geometry. Energy minimization was terminated when the root mean square gradient fell below 0.05. The initial and final energies of the protein were calculated (in kcal/mol) using the MMFF94X force field (18). The minimized structure of 2 KB3 (as shown in Figure 1) was used as the template for docking. Ten conformations were generated for each docked ligand-MA B complex. Additionally, docking consistency was confirmed by performing re-docking experiments of the co-crystallized ligand into the active site, attaining a Root Mean Square Deviation (RMSD) of less than 2 Å, which confirmed the accuracy of the docking protocol. Visualization of docked complexes was carried out using both 2D and 3D interaction maps to highlight the molecular contacts of benzene and fluoxetine with MAO-B.

### Statistical analysis

Data were tabulated in Excel, in which descriptive analysis was carried out. Data were statistically analyzed using SPSS software. The chi-squared test was used to determine the difference between socioeconomic and demographic category variables. A stepwise-linear regression analysis was used to predict the concentration of benzene in the blood of healthcare workers. Stepwise regression was used for exploratory analysis; however, its limitations—including potential bias, overfitting, and lack of diagnostic checks for residual normality, multicollinearity, and homoscedasticity—may affect the accuracy and generalizability of the reported associations.

Statistical significance was an alpha level of *p* of < 0.05.

## Results and discussion

The demographic pattern of data is presented in Table 1. It was collected from different private and government hospitals of Rawalpindi. The data contained samples of approximately 51 health care workers, in which there were 17 (33%) nurses, 13 (26%) housekeeping staff, and 21 (41%) lab workers. The majority of health care workers were men (69%), while 31% were women. Among the 51 health care workers, 55% were married and 45% were single. The level of education among the workers was varied—15% were uneducated, 10% passed primary standard, 45% passed high school, and 20 and 10% healthcare personnel had passed the college and graduate levels, respectively. A smoking habit was found in 14% health workers, while 86% were non-smokers. Job duration trend among workers was diverse, i.e., 37% workers were associated with this job for 2–5 years, while 27, 12, 6, and 18% were associated for 5–10 years, 10–15 years, 15–20 years, and 20–25 years, respectively. However, the data for work duration shows that 57% of healthcare workers spent 5–10 h daily in the hospital, while 39 and 4% workers worked daily 10–15 and 15–20 h, respectively. The monthly income of 72% workers was PKRRs 4,000–10,000, 12% had a salary of approximately PKRRs 10,000–15,000, the average monthly income of 8% was PKRRs 15,000–20,000, and the 8% had PKRRs 25,000–30,000. Those workers who were strongly satisfied with their jobs comprise 57% of the total sample, while 35% workers were satisfied, and 8% workers were dissatisfied. Nearly 71% work in shifts and 77% healthcare workers used safety equipment for protection during their duty for the prevention of any sort of injury. Nearly 37% healthcare workers received hygiene-related training. The average mean of age, height, weight and BMI of the nurses, housekeeping staff, and lab workers are presented in Table 2. The average ages of nurses, housekeeping staff, and lab workers were 32.29, 29.04, and 32.69 years, respectively. The average height of nurses, housekeeping staff, and lab workers were 5.4, 5.7, and 5.74 feet, respectively. The average weight of nurses was 61.64 kg, housekeeping staff was 61.14 kg, and lab workers was 70.53 kg. The average BMI was found to be 23.28, housekeeping staff had a BMI of 20.79, and lab workers had a BMI of 24.7.

**Table 1:** Descriptive analysis of demographic variables for surveyed population of healthcare workers (*n* = 51).

Variables	Categories	Frequency	Percentage
Profession	Nurses	17	33%
	Lab workers	13	26%
	Housekeeping staff	21	41%
Sex	Female	16	31%
	Male	35	69%
Marital status	Married	28	55%
	Single	23	45%
Education level	Uneducated	8	15%
	Primary school	5	10%
	High school	23	45%
	College level	10	20%
	University graduate	5	10%
Do you smoke?	Yes	7	14%
	No	44	86%
Job duration (years)	2–5	19	37%
	5–10	14	27%
	10–15	6	12%
	15–20	3	6%
	20–25	9	18%
Work duration (hours)	5–10	29	57%
	10–15	20	39%
	15–20	2	4%
Monthly income (PKR)	4,000–10,000	37	72%
	11,000–15,000	6	12%
	16,000–20,000	4	8%
	Above 20,000	4	8%
Job satisfaction	Strongly satisfied	29	57%
	Satisfied	18	35%
	Dissatisfied	4	8%
Work in shifts	Yes	36	71%
	No	15	29%
Use of safety equipment for protection	Yes	39	77%
	No	12	23%
Received any hygiene related training during job	Yes	19	37%
	No	32	63%

**Table 2:** Categories of health care workers and their characteristics (mean ± SD).

Demographics	Profession			*p*-value (*t*-test)		
	Nurses (17) ^a^	Housekeeping staff (21) ^b^	Lab workers (13) ^c^	a*b	b*c	a*c
Age (yrs)	32.29 ± 13.1	29.04 ± 10.8	32.69 ± 8.5	0.41	0.31	0.92
Height (feet)	5.4 ± 0.26	5.7 ± 0.36	5.64 ± 0.38	0.004	0.55	0.04
Weight (kg)	61.6 ± 11.93	61.14 ± 8.27	70.53 ± 17.12	0.87	0.03	0.10
BMI	23.28 ± 3.86	20.79 ± 2.91	24.7 ± 7.26	0.027	0.032	0.49

^a,b,c^shows the significant interaction among those 3 variables.

In Table 3, the association between profession and different socio-demographic variables is presented. The chi–squared test was used to analyze the association between profession and different variables such as job duration, work hours, monthly income, job satisfaction, sleep disturbance, and having received any hygiene-related training. Monthly income and the HCWs who work in shifts are significantly associated with their profession (*p* < 0.005). The monthly income of HCWs is statistically significant with their profession. Healthcare workers who work in shifts are strongly linked to their profession. Other variables like sleep disturbance of HCWs and having received any hygiene-related training of workers were also significant and associated with profession (*p* < 0.05). The training of workers related to hygiene was also associated with their profession. Job duration (years), monthly income, and job satisfaction were not significantly associated with the profession of HCWs.

**Table 3:** Job and personnel characteristic of selected healthcare workers.

Variables	Profession/Staff			*χ* ^2^	*p*-value
	Nurses	Lab workers	Housekeeping staff		
Job duration (years)					
2–5	7 (37)	3 (16)	9 (47)	9.82	0.278
5–10	2 (14)	5 (36)	7 (50)		
10–15	1 (17)	3 (50)	2 (33)		
15–20	2 (67)	1 (33)	0 (0)		
20–25	5 (56)	1 (11)	3 (33)		
Work hours (h)					
5–10	11 (38)	7 (24)	11 (38)	3.37	0.498
10–15	6 (30)	6 (30)	8 (40)		
15–20	0 (0)	0 (0)	2 (100)		
Monthly income × 10 ^3^					
4–10	10 (27)	6 (16)	21 (57)	25.64	<0.01
10–15	1 (17)	5 (83)	0 (0)		
15–20	2 (50)	2 (50)	0 (0)		
25–30	4 (100)	0 (0)	0 (0)		
Are you satisfied with your job?					
Strongly satisfied	11 (38)	6 (21)	12 (41)	2.18	0.702
Satisfied	4 (23)	6 (33)	8 (44)		
Dissatisfied	2 (50)	1 (25)	1 (25)		
Do you work in shifts					
Yes	17 (42)	7 (19)	12 (33)	10.67	0.005
No	0 (0)	6 (40)	9 (60)		
Sleep disturbance					
Yes	5 (71)	2 (29)	0 (0)	6.90	0.032
No	12 (27)	11 (25)	21 (48)		
Got any hygiene related training					
Yes	11 (58)	4 (21)	4 (21)	8.69	0.013
No	6 (19)	9 (28)	17 (53)		

This study primarily focused on three professions in the healthcare settings, i.e., staff nurses, lab workers, and housekeeping staff, who were expected to have routinely undergone exposure to a variety of chemical products containing benzene. The sociodemographic features of these workers helped us to identify the most significant predictors of exposures in their lifetime. It is clear from our findings that, in healthcare settings, the nature of the job and gender were the parameters that had a closed association with exposure risk. Female workers who were the most susceptible to exposure, especially, nursing staff who were predominantly women, seemed to face more exposure to benzene than the other two occupations. The use of cleaning products and sterilizing agents are sources of exposure to benzene among nursing staff. Such solvents also contain chloroform, methanol, and gasoline (15). In the general population, solvent exposure is very common and ubiquitous. Nurses, due to their close contact with patients, come in contact with pathogens often (Centers for Disease Control and Prevention 2003). Pregnant nurses may be at risk of exposure to teratogenic chemicals, which can be fatal to the fetus. Occupational exposure to both teratogenic and pathogenic chemicals is a serious cause of concern to such healthcare workers (15). In this study, there was no significant difference between the job duration of the three occupational groups. However, almost all the nurses were working in shifts. The percentage of working in shifts was highest among nurses (*p* < 0.001), which showed that they were under more stress, a consequence of which is disturbance in sleep, which was also different among them than other two occupational groups (*p* = 0.03). The results of our benzene data showed that staff nurses also had higher levels of blood benzene than housekeeping staff, and this difference was significant (*p* = 0.04; Table 3). The exposure was even higher for nurses than laboratory workers, although this difference was non-significant. One of the possible reasons behind this higher exposure may be the substandard hygiene, which prevails in most of the government hospitals. A good hygienic practice at such workplaces is necessary to minimize the accumulation of contaminants and subsequent exposure risks (19). In government hospitals, poor hygiene is prevalent, whereas in private hospitals, workers bear work overload and earn relatively lower salaries. According to NIOSH, sources of exposure among such workers include all those objects that come in contact with hospital staff in the workplace, including self-protecting equipment, etc. (15). Moreover, clothing is also a source of exposure. In particular, the contamination of workplace chemicals that may be carried to the home can be controlled by changing clothes before going home. Such clothing must also be laundered separately (20). Healthcare workers, especially nurses, face occupational exposure to organic solvents, which are commonly used for the purpose of sterilization and also laboratory chemicals. Among these organic chemicals, acetone, chloroform, benzene, and methaonil are common. Most of such chemicals are teratogenic (15). During maternity, exposure to such organic solvents can cause abortion or premature birth (21, 22). Some of the chemicals have recently come into the knowledge, and their long-term effects on health are poorly understood (23) and thus need further investigation. The healthcare workers can reduce the exposure risk by wearing solvent-resistant gloves and self-protective cloths, improving workplace ventilation (15). According to Xelegati et al. (24, 25), nurses in a hospital environment are exposed to a host of chemicals, and possible sources of these chemicals reported by them are handling of detergents, inhalation of anesthetic gasses, and exposure to formaldehyde and glutaraldehyde vapors. Benzene is an important chemical of exposure. In this survey, 56.6% nurses identified benzene as an unimportant hazardous substance of exposure. The symptoms of complex chemical exposure were reputedly the skin problems, hair fall, and immunological function disturbances. Among the five most mentioned substances, benzene was one of them.

Although staff education should reduce the exposure risk among health care workers (15), it was observed that nurses had the highest ratio of having received hygiene-related training than the other two occupations (*p* = 0.013, Table 3). It is also clear from our results that those healthcare workers who had received more health care training had more exposure to benzene. Although these results seem to be paradoxical, it may be due to the load of responsibility assigned to such professionals, who might have rendered them negligent toward hygiene-related issues because of work overload. Moreover, long hours of working in shifts cause an increase in exposure to organic solvents. Again, nurses were the most vulnerable from this point of view. There is an interaction between work stress and exposure to benzene (26). Our female subjects (nurses) also had a higher likelihood of having been afflicted with disease (*p* = 0.03). These facts again show that either nurses show reckless behavior toward self-protection, probably because of shifts in work hours, or because of sleep disturbance, which is responsible for the lack of attention and self-protection. Night shift duties and the long working hours are also associated with spontaneous abortion among nurses (27). Therefore, it is clear that nursing staff have to face more stress and sleep disturbance due to the nature of their duties. There may be a need for proper ventilation systems in the workplace to minimize the exposure risks and the need for strict use of self-protective equipment (15). According to MCMurry et al. (28), the metabolism of benzene is also influenced by age. There is an interaction between work stress and exposure to benzene (26).

The relationship between the general occurrence of disease and different variables (gender, profession, smoking habit, job duration (years), work hours (h), frequency of medical check-up, and the use of safety equipment for protection) associated with occupation was found using the chi–squared test, as presented in Table 4. Job duration (years) and gender were significantly related to the status of healthcare worker who are suffering from any disease due to their job (*p* < 0.05). Gender health is also significantly related to the disease endure of HCWs. The frequency of the medical check-up was also significantly related to the HCW disease (*p* < 0.005).

**Table 4:** Characteristic of surveyed population in hospitals in relation to response variable “have you developed any prolonged illness/sickness in the past 6 months?”

Variables	Yes	No	*χ* ^2^	*p*–value
Gender				
Women	6	10	4.73	0.03
Men	4	31		
Profession				
Nurse	6	11	4.15	0.125
Lab workers	2	11		
Housekeeping staff	2	19		
Smoking habit				
Yes	1	6	0.14	0.703
No	9	35		
Job duration (Yrs)				
2–5	1	18	12.31	0.015
5–10	1	13		
10–15	2	4		
15–20	2	1		
20–25	4	5		
Work hours				
5–10	8	21	2.83	0.243
10–15	2	18		
15–20	0	2		
Freq. of medical checkup				
No	2	24	13.91	0.001
Yearly	4	16		
Quarterly	4	1		
Use of self safety equipments				
Yes	7	32	0.28	0.591
No	3	9		

Table 5 shows the mean concentration of benzene in the blood of healthcare workers of different categories. The mean concentration of benzene in nurses was 1.43 ppm, and its range was from 0.004–6.26 ppm; the mean concentration of benzene in lab workers was 1.04, and its range was 0.002–4.74; and the mean concentration of benzene in housekeeping staff was 0.89, and its range was 0.004–4.75. Among nurses, the concentration of benzene was high as compared with other categories.

**Table 5:** Concentration of benzene in different categories of health care workers.

Variables	Freq	Benzene	Range	*p*-value
Profession				
Nurses ^a^	17	0.188	0.034–6.26	_a*b_ 0.48
Lab workers ^b^	13	0.05	0.002–1.8	_a*c_ 0.04*
Housekeeping staff ^c^	21	0.012	0.004–1.89	_b*c_ 0.48
Received any hygiene training				
Yes	18	0.61	0.004–6.26	0.008
No	31	0.013	0.002–4.75	
Gender				
Male	35	0.327	0.002–1.89	0.02
Female	16	0.356	0.44–6.2	
Smoker				
Yes	6	0.39	0.006–1.89	0.119
No	45	1.18	0.002–6.26	

* *p* < 0.05, Values showing median concentration (ppm) in blood samples (*n* = 51).

^a,b,c^shows the significant interaction among those 3 variables.

The results revealed that, in addition to the gender, the duration of the job (years) was also significantly related to the illness among healthcare workers who were exposed to organic solvents, possibly containing benzene during work. Our findings, therefore, revealed serum benzene concentration in fairly high concentrations as compared to commonly reported chemical exposure investigations, such as Swaen et al. (29), where mean concentration of benzene in the blood of Dow employees was 0.22 ppm, ranging from 0.01 ppm to 1.85 ppm.

Occupational exposure to hazardous chemicals such as benzene still occurs within both occupational and environmental settings. In developed countries, exposure of people to chemicals is often from the urban environment, where benzene is a ubiquitous pollutant released from motor vehicles (30). Environmental exposures to benzene in the general population are predominantly through inhalation due to its volatile character (31).

There is a limited number of research works available on the chemical exposure assessment to the healthcare worker, but in Pakistan, it is almost non-existent (32), although awareness regarding individual exposure to occupational and environmental chemicals is increasing (26). Healthcare workers are exposed to a variety of occupational abuses that cause serious and long-term adverse health consequences (23). Such exposure occurs mainly through inhalation, ingestion, and absorption through the skin or mucous membranes. The most common hazards occurred in hospitals due to exposure to disinfectants (33).

A stepwise linear regression model was used to predict the concentration of benzene in the blood of healthcare workers (Table 6), and the initial model can be stated as:


$$Y=b+b_{1}X_{1}+b_{2}X_{2}$$

**Table 6:** Stepwise linear regression for the prediction of benzene concentration in the blood of healthcare workers.

Model	Coefficients		*p*–value
	Unstandardized (B)	Standardized (β)	
(1) Constant	−2.520		0.007
BMI	0.147	0.473	0.000
(2) Constant	−2.444		0.000
BMI	0.061	0.196	0.034
Job duration	0.761	0.724	0.000

The final regression equation was modified according to the model that best fits the data as:


$$Y_{\left(\text{Benzene}\right)}=-2.444+0.061_{\left(\mathrm{BMI}\right)}+0.761_{\left(\mathrm{Job}\;\text{duration}\right)}$$

In the stepwise regression analysis, blood benzene concentration was used as the dependent variable, and finally, BMI and job duration appeared as significant predictors. In the regression model, routine exposure through blood benzene data is used as a proxy of exposure, taking various personal and occupational characteristics into account as predictors. In the final model, BMI and job duration appeared as significant predictors of blood benzene levels among health care workers. This model explains the 65% accuracy. A significant and strong association was observed between the concentration of benzene in blood and BMI, which indicates that BMI can be a useful predictor for the determination of benzene exposure. Our results indicated that a rise in blood benzene concentration and BMI had a positive association because of its tendency to bioaccumulate in fatty tissues (34). In our study, BMI appeared as a strong predictor. As far as the BMI of lab workers is concerned, it was significantly higher than that of housekeeping staff and also higher than that of nursing staff. It may imply the nature of the job because lab workers have limited movement during their duty hours. Their BMI might have increased due to the sedentary nature of their work, but this also indicates a possible future risk of bioaccumulation of contaminants.

**Figure 2:**
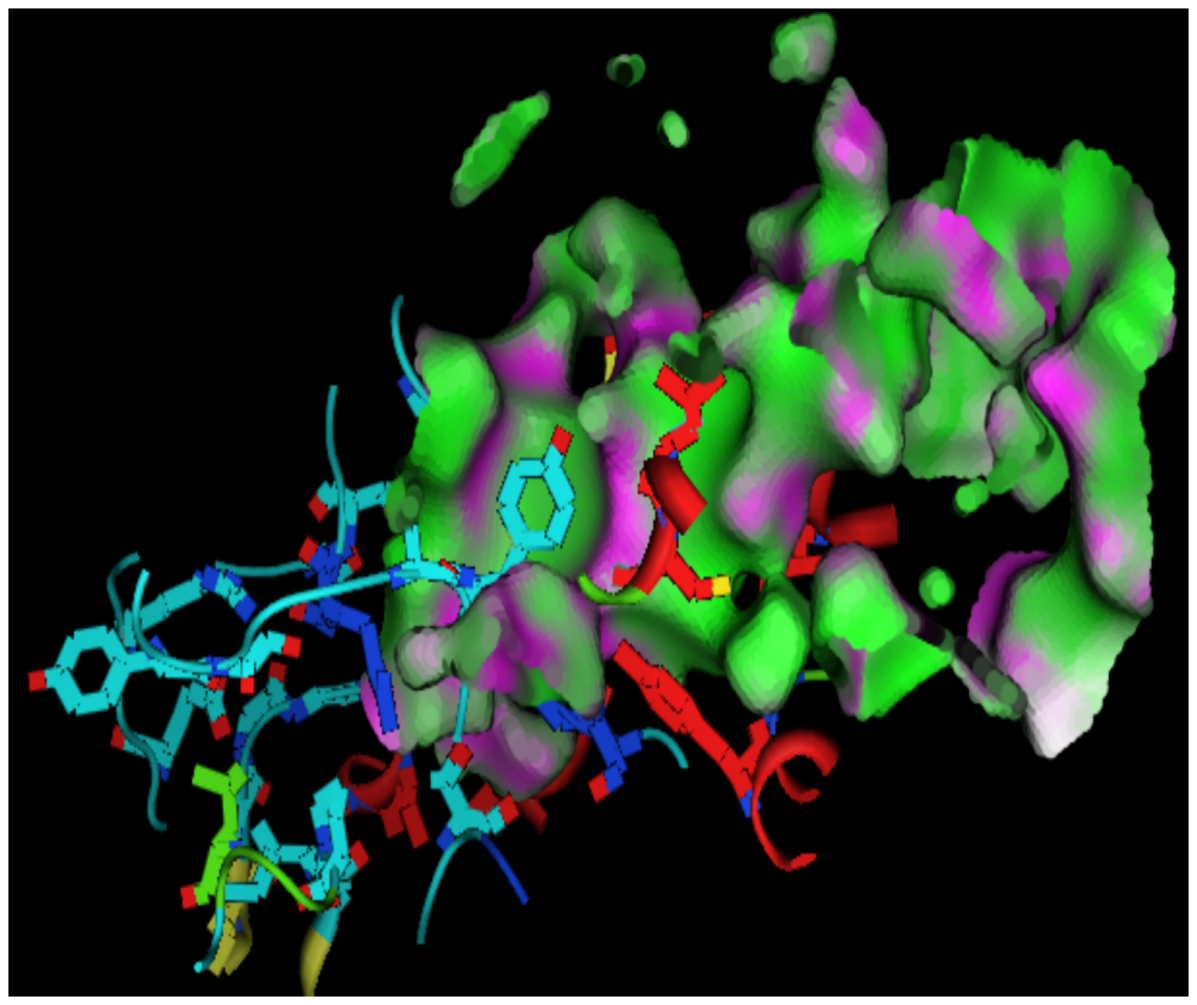
Active site of antidepressant target for Molecular modeling.

**Figure 3:**
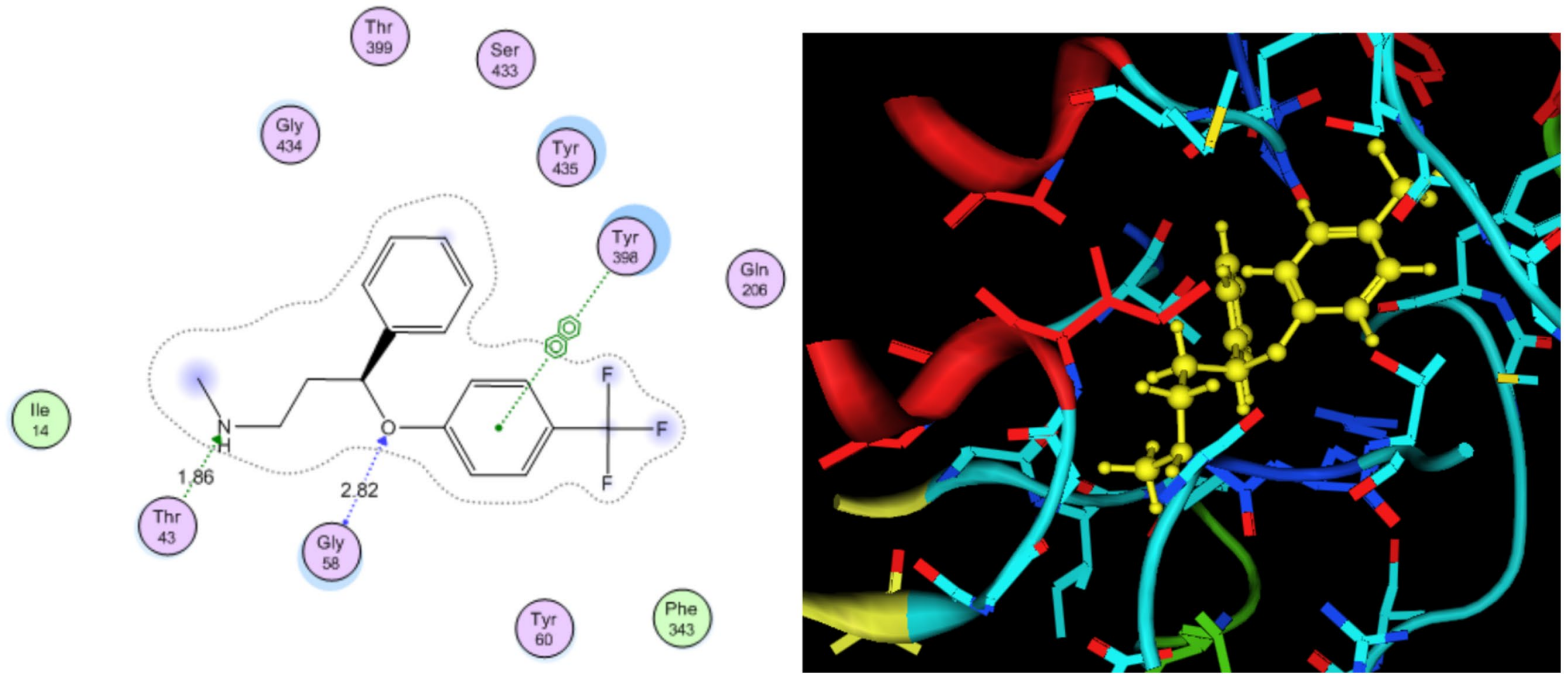
2D and 3D docked pattern of standard antidepressant Fluoxetine within the proximity contour of the target.

**Figure 4:**
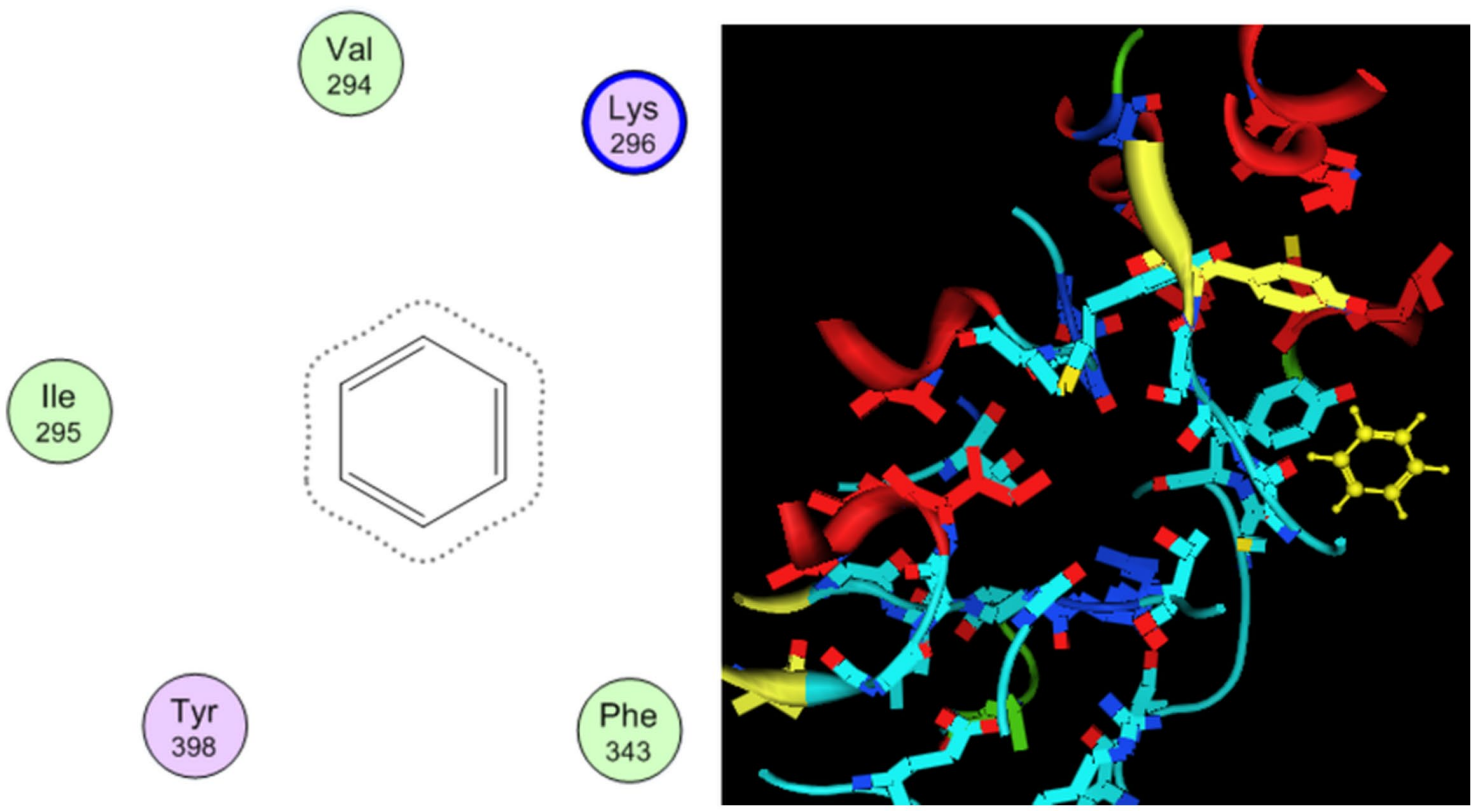
2D and 3D docking behavior of benzene in the active site of the target.

Molecular docking behavior of benzene was compared with that of the standard antidepressant Fluoxetine. The 3D structure of the target (PDB ID: 2BK3) was extracted and prepared for docking, as shown in Figure 1. The active site was analyzed by the site finder tool of MOE (35), as shown in Figure 2. Ligand preparation was performed for benzene and the standard antidepressant fluoxetine. Both ligands were energy-minimized to their lowest-energy conformations before molecular docking. 2D and 3D docked poses of both compounds with the targets are shown in Figures 3, 4. For each ligand, 10 conformations were generated and scored using the London dG scoring function, followed by refinement with GBVI/WSA dG.

The hazardous effect of benzene on health was also studied using molecular docking studies, which showed that benzene is one of the sources of anxiety and depression in the body. Antidepressant fluoxetine was used as a standard for analysis of the binding pattern of benzene with the target. Fluoxetine showed strong hydrogen binding and *π*-π interactions with Tyr398 and Gly58. The docked binding energy of fluoxetine was −11.04 kcal/mol, and that of benzene is −6.63 kcal/mol.

This showed that the benzene–target complex has an unfavorable conformation for dopamine production, as it does show binding in the active site, such as antidepressant fluoxetine. Benzene exhibited weak hydrophobic and π–π interactions within the catalytic pocket, with a docking score of −6.63 kcal/mol. Although benzene lacks strong hydrogen bonding, its binding indicates a possible interference with MAO-B function, potentially disrupting dopamine metabolism. These findings recommend that benzene contact may exert neurotoxic effects by binding non-specifically to MAO-B, thereby altering neurotransmitter regulation and prompting individuals to depression and anxiety. While its binding affinity is much lower than that of fluoxetine, chronic low-level exposure could contribute cumulatively to adverse neurological outcomes (36, 37) (Table 7).

**Table 7:** Binding interactions and binding score of docked compounds.

Compounds	Key interactions	Residues	Dock score Kcal/mol
Benzene	Hydrogen bonding, π–π stacking, hydrophobic	Val294, Ile295	−6.63
Fluoxetine	Hydrophobic, weak π–π stacking	Tyr398 and Gly58, Ile14	−11.04

## Conclusion

This study highlights the significant occupational exposure to benzene among healthcare workers, particularly nurses, due to prolonged work hours, inadequate hygiene practices, and limited use of protective measures. A cumulative risk over time is indicated by the positive correlation between benzene levels and variables like BMI and the length of employment. *In silico* molecular docking studies show that benzene is one of the sources of conformational changes of MAO B and is responsible for depression and anxiety, and it has neurotoxic potential and raises the possibility of long-term neurological effects. It is recommended that occupational health programs should prioritize primary prevention in exposure assessment and monitoring. As a result, ongoing monitoring appears to be essential for evaluating environmental hygiene conditions as well as lowering exposure risks in hospital premises. Organizations should employ the hierarchy of controls to lower exposure among healthcare workers. Implementing well-designed systems that offer routine medical tests and data to support preventative measures and guarantee early diagnosis and treatment of occupation-related disorders is one way to enhance health surveillance. To reduce health hazards, it is also necessary to streamline work hours and enhance workplace hygiene. Hospitals and healthcare employers must prioritize workplace safety and mental health by enforcing protective policies, ensuring adequate resources, and fostering a culture of support.

## Data Availability

The raw data supporting the conclusions of this article will be made available by the authors, without undue reservation.

## Footnotes

^1^ www.rcsb.org/pdb
